# Construction of Nontoxic Polymeric UV-Absorber with Great Resistance to UV-Photoaging

**DOI:** 10.1038/srep25508

**Published:** 2016-05-03

**Authors:** Zhong Huang, Aishun Ding, Hao Guo, Guolin Lu, Xiaoyu Huang

**Affiliations:** 1 Key Laboratory of Synthetic and Self-Assembly Chemistry for Organic Functional Molecules, Shanghai Institute of Organic Chemistry, Chinese Academy of Sciences, 345 Lingling Road, Shanghai 200032, People’s Republic of China; 2 Department of Chemistry, Fudan University, 220 Handan Road, Shanghai 200433, People’s Republic of China

## Abstract

In this article, we developed a series of new nontoxic polymeric UV-absorbers through covalently attaching a benzophenone derivative onto the main chain of poly(vinyl chloride) (PVC) via mild and quantitative click chemistry. Azide groups were firstly introduced into the backbone of PVC via a nucleophilic reaction without affecting polymeric skeleton. Copper-catalyzed Husigen-Click cycloaddition reaction was performed between the pendant azide groups of PVC and alkynyl of (2-hydroxy-4-(prop-2-ynyloxy)phenyl)(phenyl)methanone at ambient temperature for affording the desired PVC-based UV-absorbers (PVC-UV) with different amounts of benzophenone moieties, which displayed great resistance to photoaging without degradation while exposed to UV irradiation. These polymeric UV-absorbers also showed good solubilities in common organic solvents and no cytotoxicity *vs*. HaCat cell. Small amounts of PVC-UV were homogeneously mixed with PVC as additive for stabilizing PVC against UV-photoaging without degradation and releasing small molecule even after 200 h while keeping thermal stability. This route of polymeric additive clearly paved an efficient way for solving the puzzle of separation of small molecule additive.

Poly (vinyl chloride) (PVC) is a kind of extensively used thermoplastic material due to its valuable properties such as superior mechanical and physical properties, high chemical and abrasion resistance. PVC has been widely utilized in durable applications including pipe, window profile, house siding, wire cable insulation, and flooring^1^,^2^,^3^. It stands second in the world just after polyolefins, concerning the production and consumption of a synthetic material^4^. However, when exposed to ultraviolet (UV) irradiation, PVC is susceptible to photochemical decomposition because it contains abnormal structures originating from synthesis^5^; this deficiency obviously limits its use in outdoor applications. In the radical polymerization of vinyl chloride, a number of isomeric forms and structural defects resulted from the formation of the main chain. These anomalous structures could induce photo- instability during the usage since that photodecomposition of PVC often begins with internal allylic chloride and tertiary chloride structural defects in the main chain^5^. Because of the degradation, the processing and mechanical properties of PVC are certainly worsened. Therefore, studies on the inferior photostability are of vital importance to the users of PVC.

Currently, small molecule UV-stabilizers are usually added by physical mixing for inhibiting the photodegradation of PVC. Rigid PVC compositions for outdoor application prefer the usage of titanium dioxide (TiO_2_) or some light-fast organic pigments because of their excellent ability in UV screening^6^, a relatively rare usage option of UV-absorber. Meanwhile, for flexible PVC, especially transparent PVC products, UV-absorbers are typically added to protect the material from discoloring as well as surface cracking during long-term exposure to sunlight^7^. These UV-absorbers include a comprehensive range of compound types, but the ones most commonly used in industry now are the benzophenone and benzotriazole classes, which work by absorbing most of incident UV radiation and then dissipating the energy in a nondestructive form without releasing a small molecule as shown in Fig. 1,^8^.

The benzophenone classes have advantages of cheap price, simple processing of industrial production, excellent photostability, less discoloring, and good water resistance etc., which make them popular in PVC outdoor applications^11^. However, benzophenone UV-absorbers are usually easy to separate out or be extracted by solvents due to its low molecular weight and incompatibility with PVC macromolecules^12^,^13^. This disadvantage not only influences the durability of its effectiveness but can cause pollution to the environment because most of benzophenone UV-absorbers are toxic to humans or animals^14^,^15^.

In order to meet the technical, environmental, and economic requirements of high performance polymer, the studies on UV-absorbers are becoming increasingly active in recent years for expanding PVC outdoor applications and preventing environment from deteriorating. Nowadays, most studies on UV-absorbers for PVC are as followings^16^,^17^,^18^: (1) increasing the molecular weight of UV-absorbers; (2) introducing UV-absorber moieties covalently through random copolymerization; (3) intra-molecular multifunction of UV-absorbers. Nevertheless, these modifications also lead to some new problems^12^,^19^: the light stability of UV absorbers with relatively high molecular weight (still not comparable to PVC matrix) is usually not good enough as low molecular weight species due to poor dispersion in polymer matrix; the copolymerization with UV absorbers will inevitably affect the material properties of polymers; intramolecular multifunction often has a bad effect on material application in thermal stability, compatibility, color and so on. Thus, there is still a strong demand from industrial application to develop a new approach for stabilizing PVC against photoaging without the separation of UV-absorber.

To solve the puzzle of separation of small molecule additive for PVC, especially UV-absorber, covalent attachment of UV-absorber onto the polymeric skeleton with similar chemical structure and molecular weight with matrix, i.e. PVC, may be a feasible strategy for increasing the compatibility between matrix and additive. Since the main chain of PVC possesses many reactive sites like allyl chloride and normal chloride^20^,^21^, it is possible to covalently attach small amount of benzophenone-type UV-absorber onto the backbone of PVC by nucleophilic substitution reaction. If so, this kind of modified PVC bearing benzophenone moieties not only can act as a polymeric UV-absorber for stabilizing PVC against UV-photoaging, but has good compatibility with PVC because of its almost same chemical characters so that it will not separate out or be extracted by solvents when used as an additive in PVC. Thus, we can imagine this polymeric UV-absorber linking benzophenone to PVC main chain can protect PVC products from photodegradation lastingly and effectively, without worrying about the environmental problems caused by the separation of small molecule additive.

Herein, we developed a new approach of covalently introducing benzophenone moieties into the main chain of PVC via mild and highly efficient Husigen-Click cycloaddition reaction, for constructing PVC-based UV-absorbers (PVC-UV) as shown in Fig. 2. These nontoxic polymeric additives could be homogeneously mixed with PVC matrix for protect PVC matrix from photodegradation lastingly and effectively, even after exposure to artificial UV weathering for 200 h.

## Results and Discussion

### Synthesis of PVC-based UV-absorber

The objective of this work is to develop a kind of new polymeric (PVC-based) additive (UV-absorber) compatible with the matrix (PVC), taking the place of small molecule additive for preventing the separation of small molecule additive. Therefore, linking small molecule UV-absorber covalently to the main chain of PVC via a highly efficient and mild coupling reaction is obviously a feasible approach for preparing the PVC-compatible polymeric additive. From the viewpoint of chemical structure, every repeated unit of PVC possesses a relatively active Cl atom, which can be readily substituted through a nucleophilic reaction^21^. For example, NaN_3_ could be employed to substitute Cl atom so as to introduce azide groups in the repeated units^22^,^23^. Azide group has been reported to easily react with alkynyl under mild conditions (e.g. room temperature) for quantitatively forming 1,2,3-triazole ring without emitting any small molecule^24^, i.e. Husigen 1,3-dipolar cycloaddition reaction, a most famous Click chemistry example. Thus, a possible synthetic route for constructing PVC-based UV-absorber emerged, that is to treat azido-substituted PVC with alkynyl-functionalized small molecule UV-absorber.

The first point is to functionalize small molecule UV-absorber with alkynyl while keeping its original anti-photoaging property. 2,4-Dihydroxybenzophenone was selected as mother compound for further propargylation because the hydroxyl at 4-position is very active for nucleophilic substitution reaction while other groups keep inert^25^. The nucleophilic substitution reaction occurred between the 4-position hydroxyl of 2,4-dihydroxybenzophenone and the alkynyl of 3-bromo-1-propyne to provide the desired alkynyl-functionalized derivative of 2,4-dihydroxybenzophenone, (2-hydroxy-4-(prop-2-ynyloxy)phenyl)(phenyl)methanone, with a moderate yield (75%). The chemical structure of the derivative was characterized by ^1^H NMR as shown in Fig. S1 (see supporting information). The typical resonance signal of one proton of alkynyl appeared at 2.59 ppm while the peak at 12.67 ppm was attributed to one proton of 2-position hydroxyl. The derivative is a kind of white powder (see the inset of Fig. S2B in supporting information) in comparison with the khaki powder of 2,4-dihydroxybenzophenone (see the inset of Fig. S2A in supporting information). UV-vis spectrum of the derivative (Fig. S2B in supporting information) was very similar to that of 2,4-dihydroxybenzophenone (Fig. S2A in supporting information), which implied the sustaining of the original anti-photoaging property of 2,4-dihydroxybenzophenone.

**Figure 3:**
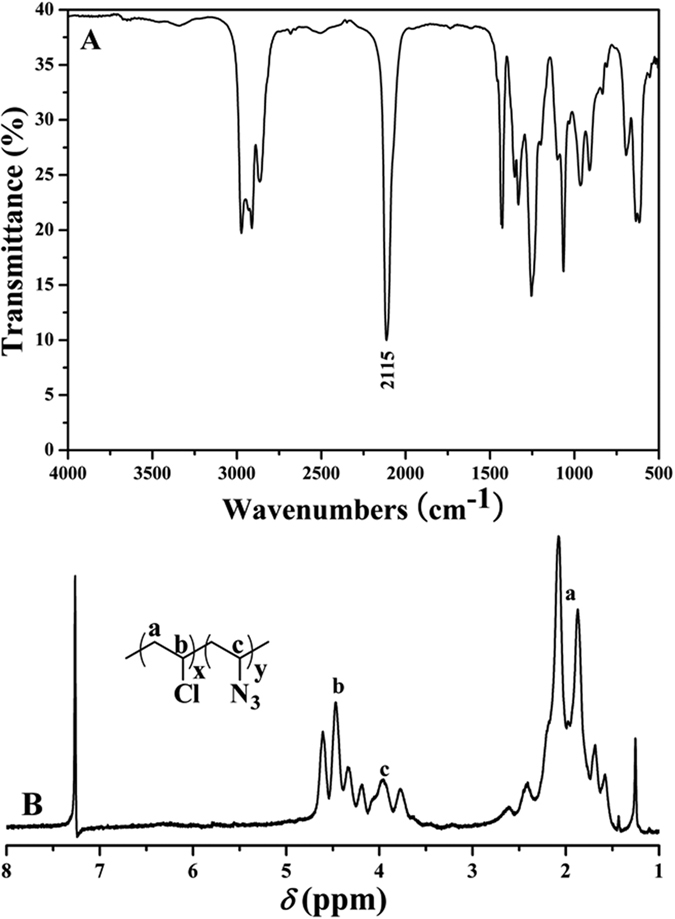
FT-IR (**A**) and ^1^H NMR (**B**) spectra of PVC-N_3_.

**Figure 4:**
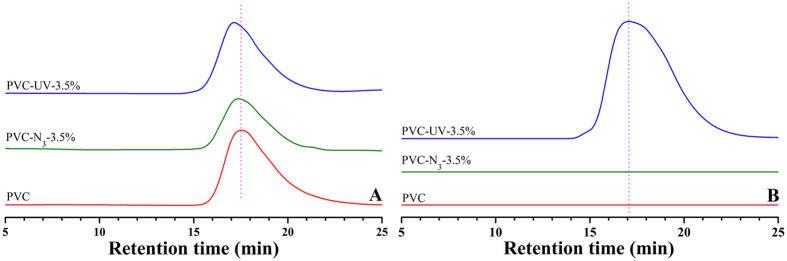
GPC curves of PVC, PVC-N_3_-3.5%, and PVC-UV-3.5% in THF using RI detector (**A**) and UV detector (**B**).

Another point is to obtain the azido-substituted PVC (PVC-N_3_) via a nucleophilic substitution reaction. NaN_3_ was used to convert Cl atom in the repeated unit of PVC to azide group for providing PVC-N_3_. FT-IR and ^1^H NMR were employed to examine the chemical structure of PVC-N_3_. FT-IR spectrum of PVC-N_3_ is shown in Fig. 3A and the characteristic peak of azide group appeared at 2115 cm^−1^, which evidenced the successful introduction of azide group. Figure 3B shows ^1^H NMR spectrum of PVC-N_3_ in CDCl_3_. The resonance signals of three protons in C*H*_2_C*H*Cl repeated unit still appeared at 1.58–2.08 ppm (peak ‘a’) and 4.34–4.60 ppm (peak ‘b’), respectively. However, new peaks at 3.78, 3.96, and 4.19 ppm (peak ‘c’) are attributed to one proton in CH_2_C*H*N_3_ repeated unit, which also demonstrated the existence of azide group. In the current case, the substitution ratio of N_3_ determined by elemental analysis (see Table S1 in supporting information) was regulated by varying the reaction time and the ratio ascended with the extending of nucleophilic substitution reaction. Thus, we obtained three PVC-N_3_ samples (PVC-N_3_-3.5%, PVC-N_3_-7.5%, and PVC-N_3_-12.9%) with different contents of azide group as summarized in Table S1 (see supporting information). Their molecular weights were all a little higher than that of PVC due to the introduction of N_3_ group while their molecular weight distributions were all very similar to that of PVC. As shown in Fig. 4A, the shape of GPC curve of PVC-N_3_-3.5% was almost same as that of PVC while with similar molecular weight and polydispersity. All aforementioned results clearly witnessed the successful introduction of azide groups into PVC without affecting polymeric skeleton.

The following critical step was the coupling reaction between the alkynyl of (2-hydroxy-4-(prop-2-ynyloxy)phenyl)(phenyl)methanone and the pendant azide groups of PVC-N_3_ for affording the target product, PVC-based UV-absorber (PVC-UV). This 1,3-dipolar cycloaddition reaction was performed in THF at ambient temperature using CuBr/PMDETA as catalytic system^26^,^27^. All three PVC-N_3_ samples (PVC-N_3_-3.5%, PVC-N_3_-7.5%, and PVC-N_3_-12.9%) were employed for Husigen- Click cycloaddition reaction so as to afford three PVC-UV samples, which were designated as PVC-UV-3.5%, PVC-UV-7.5%, and PVC-UV-12.9%, respectively. After Husigen-Click cycloaddition reaction, the molecular weight of every PVC-UV sample was higher than that of the corresponding PVC-N_3_ sample (see Table S2 in supporting information) because the molecular weight of benzophenone moiety (252) was relatively high; nevertheless, its molecular weight distribution was still very similar to that of PVC and PVC-N_3_. The shape of GPC curve of PVC-UV-3.5% was also very similar to that of PVC and PVC-N_3_-3.5% (Fig. 4A) while keeping similar molecular weight distribution. GPC curve was also monitored by UV detector with a detection wavelength of 276 nm since that (2-hydroxy-4-(prop-2-ynyloxy)phenyl) (phenyl)methanone had a strong UV absorption peak around 276 nm (see Fig. S2B in supporting information). As shown in Fig. 4B, GPC curves of PVC and PVC-N_3_-3.5% were both a straight line without any elution peak; however, PVC-UV-3.5% showed a strong elution peak with a similar shape and retention time compared to that monitored by RI detector. This fact strongly supported the presence of benzophenone moiety in the repeated unit of PVC.

**Figure 5:**
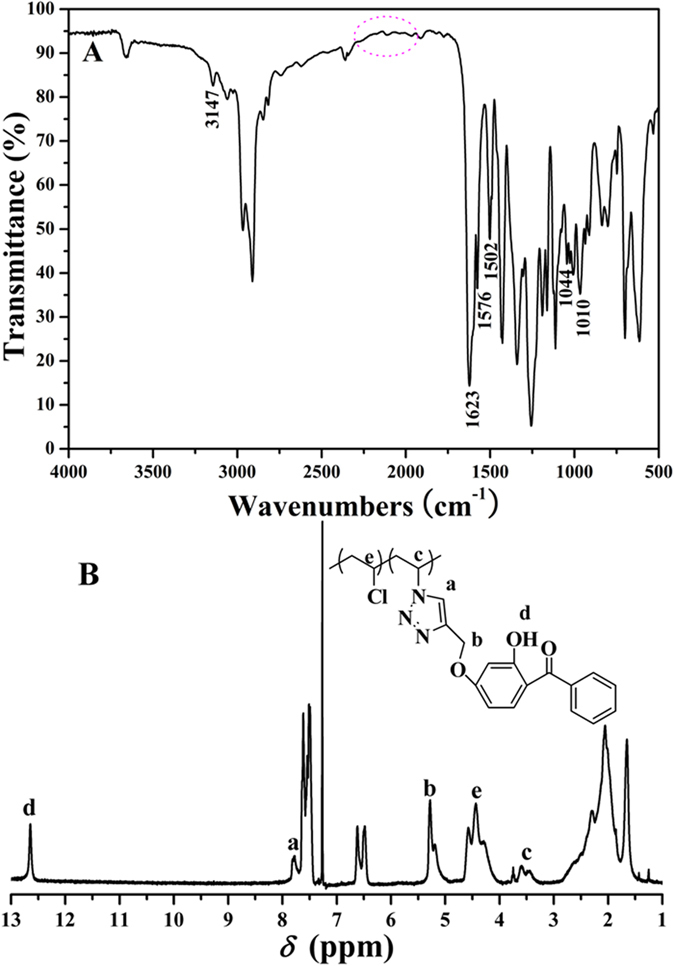
FT-IR (**A**) and ^1^H NMR (**B**) spectra of PVC-UV.

The product of Husigen-Click cycloaddition reaction, PVC-UV, was characterized by FT-IR and ^1^H NMR. FT-IR spectrum after the reaction (Fig. 5A) showed the disappearance of typical signal of azide group around 2115 cm^−1^ while a series of new peaks originating from triazole ring (3147 (=C-H), 1623 (-C=C-), and 1044 (C-N-) cm^−1^)^28^ and phenyl (1576 and 1502 cm^−1^) appeared, which verified the occurrence of cycloaddition reaction and the formation of 1,2,3-triazole ring. Furthermore, the peak at 1010 cm^−1^ affirmed the ether linkage between 1,2,3-triazole ring and phenyl of benzophenone moiety. The existence of phenyl of benzophenone moiety was also illustrated by the resonance signals at 6.49, 6.63, 7.51, and 7.62 ppm in ^1^H NMR spectrum (Fig. 5B). The peak at 12.65 ppm (peak ‘d’) belonged to one proton of 2-position hydroxyl of benzophenone moiety. The minor peak at 7.79 ppm (peak ‘a’) was originated from one proton of C*H* in 1,2,3-triazole ring, this indicating the covalent 1,2,3-triazole ring linkage between PVC backbone and benzophenone moiety. The peaks at 3.46, 3.60 (peak ‘c’), 4.29, 4.44, and 4.57 (peak ‘e’) ppm corresponded to two protons of C*H*_2_CHN and C*H*_2_CHCl, respectively, which showed the co-existence of common CH_2_CHCl and new CH_2_CH-triazole repeated units in PVC-UV. The ether linkage between 1,2,3-triazole ring and phenyl was also proved by the signals at 5.20 and 5.29 ppm (peak ‘b’). *T*_g_ of PVC-UV was measured by DSC (see Table S2 in supporting information) and the values of three samples (81–83 ^o^C) were all slightly higher than that of PVC (78.22 ^o^C) due to the incorporation of rigid triazole and benzene rings. The values rose with the increasing of the content of benzophenone moiety. Moreover, the solubility of PVC-UV is similar to that of PVC and it is soluble in common organic solvents, this implying good processibilty. Now, we can conclude from the above- mentioned points that benzophenone moiety was covalently attached to the backbone of PVC via Husigen-Click cycloaddition reaction with keeping polymeric skeleton.

### Photoaging and cytotoxicity of PVC-UV

The product of Husigen-Click cycloaddition reaction, PVC-UV, was supposed to be stable while exposed to UV irradiation due to the incorporation of benzophenone moiety. It has been reported that PVC is very sensitive to 310 nm UV light^29^,^30^ so that we chose a UV-aging box (110 W) with a 313 nm UV lamp as the light source. The light aging process was monitored by checking the color change and measuring FT-IR every 10 h. To compare the photostability, both PVC and PVC-UV were taken for ultraviolet aging test at 20 ^o^C.

**Figure 6:**
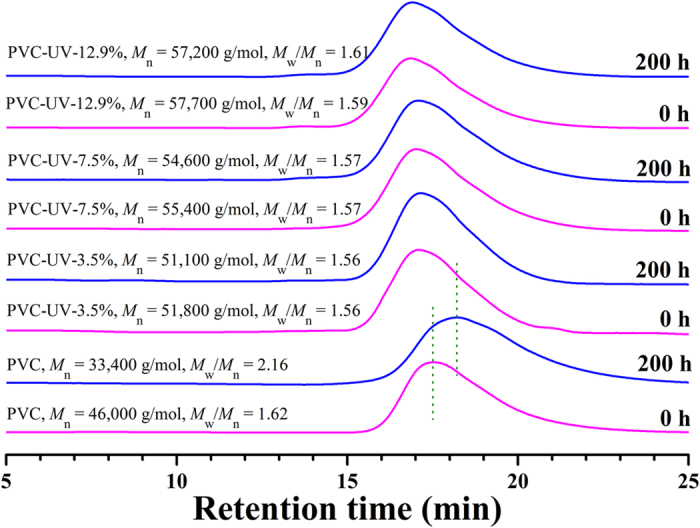
GPC curves of PVC and PVC-UV in THF before and after UV irradiation.

For pure PVC, it can be clearly seen from Fig. S3A (see supporting information) that its color gradually changed from white (0 h, before UV irradiation) to yellow (200 h after UV irradiation). The darkened color indicated the photodegradation, which generated carbonyls by photooxidation and polyenoid by eliminating HCl, along with the crosslinking and cleavage of polymer chains^31^. Figure 6 shows GPC curves of pure PVC before and after UV irradiation. The molecular weight of PVC (*M*_n_) fell sharply from 46,000 g/mol to 33,400 g/mol after 200 h of UV irradiation and the molecular weight distribution (*M*_w_/*M*_n_) broadened from 1.62 to 2.13, which distinctly verified the cleavage of PVC chains.

Carbonyls are usually used as the main index of photodegradation^32^,^33^. The generation of carbonyls certainly led to the appearance of a new peak around 1700 cm^−1^ in FT-IR spectrum, which was absent in pure PVC before UV irradiation. Therefore, carbonyl index, i.e. photoaging speed, is defined as the integration area ratio of carbonyl peak (around 1700 cm^−1^) to methylene peak (around 1430 cm^−1^)^33^. In the current case, there was not a peak around 1700 cm^−1^ in FT-IR spectrum of PVC before UV irradiation as shown in Fig. S3B (see supporting information). After UV irradiation at 313 nm, a new peak appeared at 1719 cm^−1^ and the intensity of the peak increased with the extending of irradiation time (see Fig. S3B in supporting information). Herein, carbonyl index is defined as the integration area ratio of carbonyl peak (1719 cm^−1^) to methylene peak (1427 cm^−1^) and its dependence on UV irradiation time is plotted in Fig. S4 (see supporting information). The carbonyl index of pure PVC monotonously rose throughout the irradiation process. It was also found from Fig. S4 (see supporting information) that the photoaging speed after 100 h was obviously much faster than that before 100 h. This can be explained by the self-catalysis effect of HCl to the elimination of PVC chains, and the defects caused by UV-photoaging also auto-accelerated the photodegradation^34^,^35^. Finally, the carbonyl index of pure PVC reached 0.3331 after 200 h of UV irradiation.

**Figure 7:**
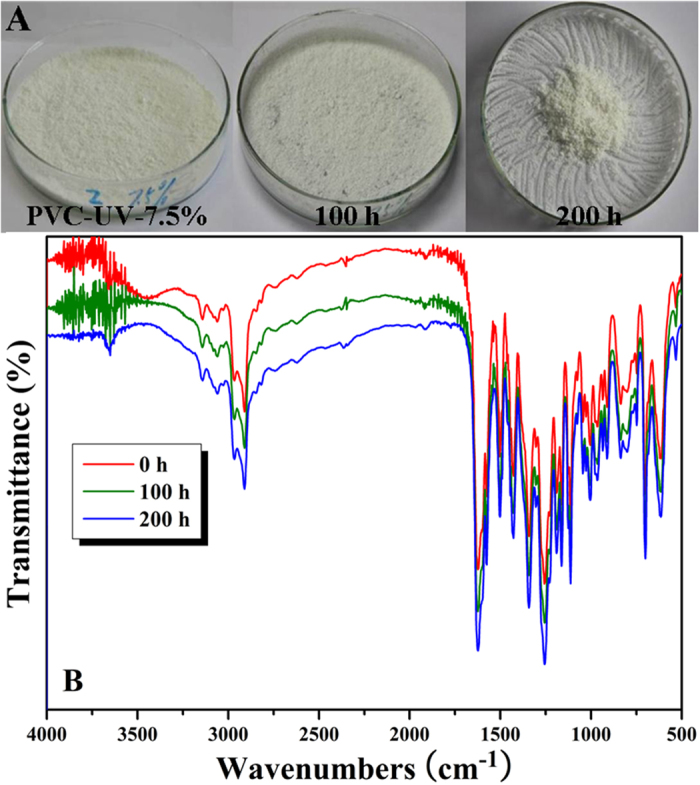
(**A**) Color changes of PVC-UV-7.5% under UV irradiation. (**B**) FT-IR spectra of PVC-UV-7.5% under UV irradiation.

However, for three PVC-UV samples with different amounts of benzophenone moieties, it was found that they all showed great resistance to UV-photoaging without releasing any small molecule according to the mechanism as shown in Scheme 1. The color of all three samples almost did not change even after 200 h of UV irradiation as shown in Figs 7A and S5A (see supporting information), and Fig. S7A (see supporting information), respectively, which was completely different from that of PVC. The molecular weights of three PVC-UV samples after 200 h of UV irradiation were all very similar to those before UV irradiation (just a minor fall in the range of 500–1000 g/mol) while keeping similar curve shape and molecular weight distribution (Fig. 6), which meant the absence of photodegradation of PVC chains.

FT-IR spectra of three PVC-UV samples are shown in Figs 7B and S5B (see supporting information), and Fig. S7B (see supporting information), respectively. Different from PVC, the spectra before and after UV irradiation were almost same and the peak of carbonyl showed no significant change during the photoaging process.

**Figure 8:**
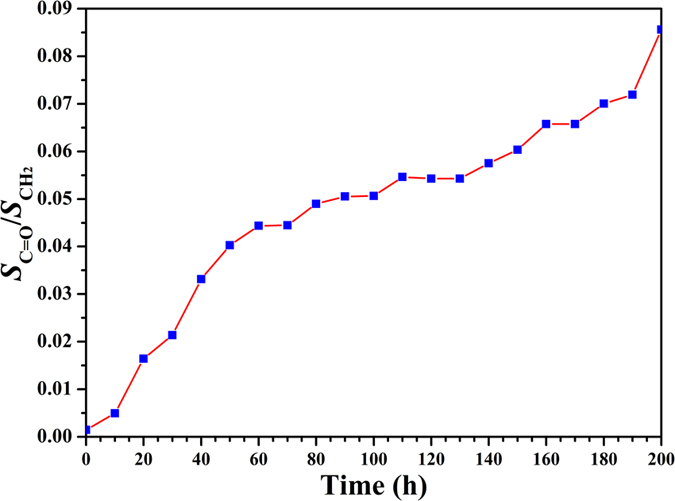
Dependence of the integration area ratio of carbonyl peak to methylene peak (*S*_C=O_/*S*_CH2_) of PVC-UV-7.5% on UV irradiation time at 20 ^o^C.

In addition, the carbonyl index of all three PVC-UV samples went up very slowly (Figs 8 and S6 (see supporting information), and Fig. S8 (see supporting information)), in comparison with that of pure PVC. The carbonyl index of PVC-UV-3.5%, PVC-UV-7.5%, and PVC-UV-12.9% reached 0.0596, 0.0856, and 0.1188 after 200 h of UV irradiation, respectively, which were much lower than that of PVC (0.3331). Though GPC curves (Fig. 6) showed the molecular weight of PVC-UV almost kept constant after UV irradiation, it is still necessary to check whether any benzophenone moiety was released via any possible cleavage of 1,2,3- triazole ring linkage during UV-photoaging process. Then, all three PVC-UV samples after UV irradiation were dissolved in THF followed by precipitating into methanol; after filtration, the filtrate was taken for UV-vis measurement. All three PVC-UV samples showed no absorption peak in the range of 200–350 nm, which indicated the absence of benzophenone moiety, i.e. stable covalent linkage between PVC and benzophenone moiety. From these results, it can be concluded that PVC-UV is stable against UV irradiation even after 200 h without releasing small molecule, accompanied by keeping polymeric skeleton, molecular weight, and polydispersity.

**Figure 9:**
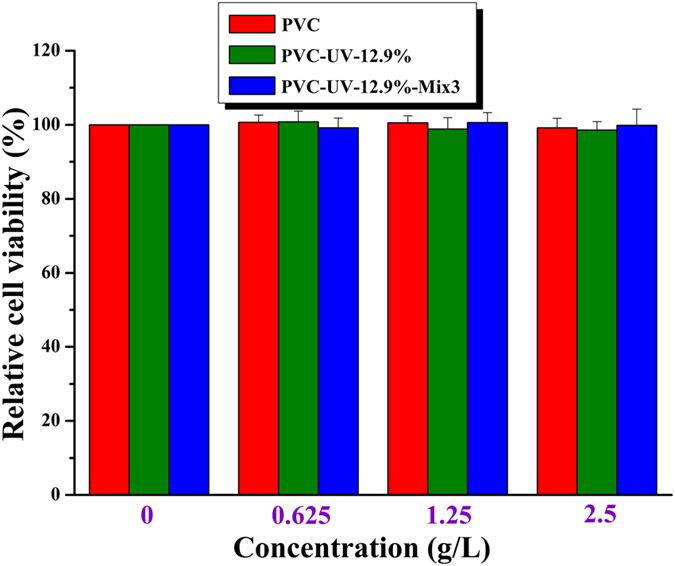
Relative cell viability of HaCat cells incubated with impregnated solutions of PVC, PVC-UV-12.9%, and PVC-UV-12.9%-Mix3 for 72 h, error bars were based on triplet samples.

PVC has been proved to be nontoxic to the skin of people^4^ so that PVC-based products are often touched by the skin of people (e.g. disposable syringe). After the incorporation of different amounts of benzophenone moiety, the cytotoxicity of PVC-UV became unknown and it must be clarified before employing it as UV-absorber for PVC matrix. Therefore, *in vitro* cytotoxicity of PVC-UV was evaluated by measuring the relative cell viability of human immortalized non-tumorigenic keratinocyte cell line HaCaT via WST assay using CCK-8. HaCaT cells were incubated with impregnated solutions of PVC-UV-12.9% and PVC for 72 h followed by measuring the absorbance at 450 nm. As shown in Fig. 9, the relative cell viability of impregnated solution with zero concentration was defined as 100% for control group. The relative cell viabilities of HaCat were always very high (>98.5%) throughout the whole concentration range (up to 2.5 g/L) for PVC-UV-12.9%, which meant that the polymeric UV-absorber, PVC-UV, has almost no cytotoxicity *vs*. HaCat cell. Combined with its great resistance to UV irradiation even for 200 h, nontoxic PVC-UV can be as deemed as potential anti-photoaging additive for PVC.

### Stabilization of PVC against UV-photoaging using PVC-UV

**Figure 10:**
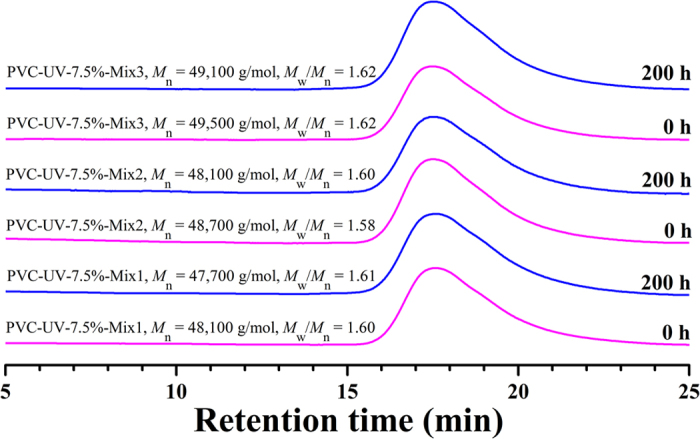
GPC curves of PVC/PVC-UV-7.5% mixtures with different formulations in THF before and after UV irradiation.

To evaluate whether PVC-UV is a suitable anti-photoaging additive for PVC, all three PVC-UV samples were mixed with PVC by dissolution-precipitation approach and the formulations were summarized in Table S3 (see supporting information). The amount of benzophenone moiety was about 1.15–3.16 wt%, which was similar to actual amount of small molecule additive for PVC-based products. All nine PVC/PVC-UV mixtures showed a sole *T*_g_ higher than that of PVC (78.22 ^o^C) but lower than that of PVC-UV; for example, *T*_g_s of PVC-UV-3.5%-Mix3, PVC-UV- 7.5%-Mix3, and PVC-UV-12.9%-Mix3 with most but same amount of benzophenone moiety were 79.35 ^o^C, 79.47 ^o^C, and 79.40 ^o^C, respectively. These sole *T*_g_s distinctly showed that all nine PVC/PVC-UV mixtures were homogeneous without microphase separation, i.e. PVC-UV is compatible with PVC. Though we employed the approach of dissolution-precipitation commonly used in chemistry laboratory to prepare the mixtures, however, it is easy for industry to compound PVC and PVC-UV because they have been proved to be compatible. Furthermore, GPC curves of all nine PVC/PVC-UV mixtures only showed an elution peak as shown in Fig. 10. The molecular weight of PVC/PVC-UV mixture was higher than that of PVC (46,000 g/mol) but lower than that of PVC-UV as summarized in Table 1 and the molecular weight distribution was very similar to that of PVC (1.62).

The *in vitro* cytotoxicity of PVC/PVC-UV mixture *vs*. HaCat cell was also evaluated via WST assay using CCK-8. It can be seen from Fig. 9 that the relative cell viability of impregnated solution with zero concentration was defined as 100% for control group. The relative cell viabilities of HaCat were always very high (>98.5%) throughout the whole concentration range (up to 2.5 g/L) for PVC-UV- 12.9%-Mix3, which implies that nontoxic PVC/PVC-UV mixture is suitable for industry application.

**Figure 11:**
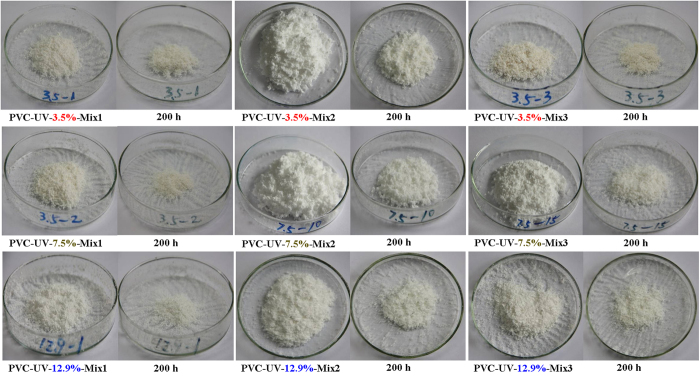
Color changes of PVC/PVC-UV mixtures with different formulations under UV irradiation.

The photostability of PVC/PVC-UV mixture was also investigated by ultraviolet aging test at 20 ^o^C. Different from PVC (see Fig. S3A in supporting information), the color of all nine PVC/PVC-UV mixtures almost kept white even after 200 h of UV irradiation (Fig. 11), this indicating the absence of photodegradation. This point was also witnessed by the almost constant molecular weights of all nine PVC/PVC- UV mixtures even after 200 h of UV irradiation (just a minor fall in the range of 400–1000 g/mol, Table 1) accompanied by the similar curve shape and molecular weight distribution (Fig. 10). These results demonstrated that PVC/PVC-UV mixtures showed great resistance to UV-photoaging without releasing any small molecule.

**Figure 12:**
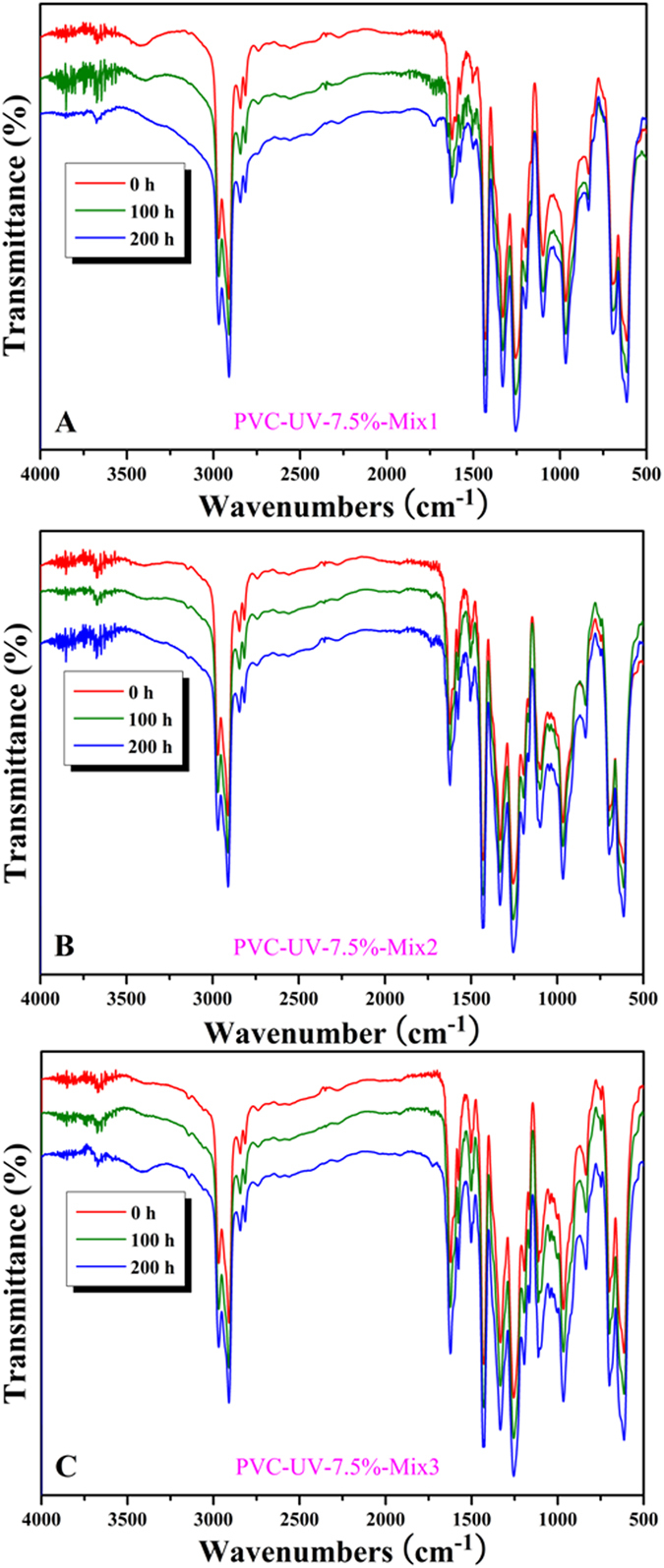
FT-IR spectra of PVC/PVC-UV-7.5% mixtures with different formulations under UV irradiation.

**Figure 13:**
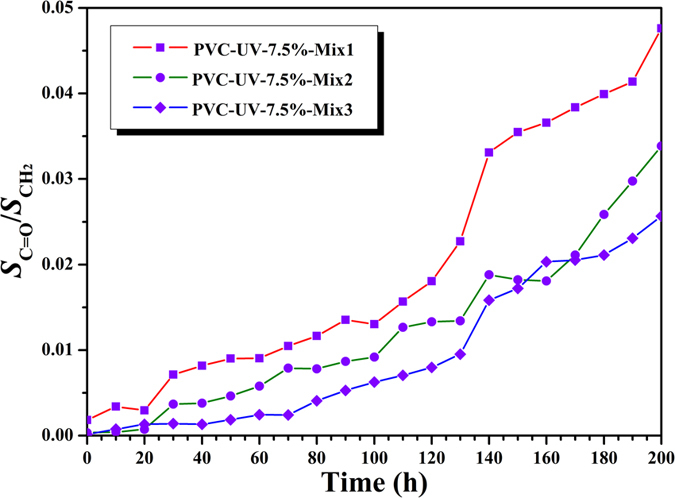
Dependence of the integration area ratio of carbonyl peak to methylene peak (*S*_C=O_/*S*_CH2_) of PVC/PVC-UV-7.5% mixtures with different formulations on UV irradiation time at 20 ^o^C.

Figures 12 and S9 (see supporting information), and Fig. S10 (see supporting information) showed FT-IR spectra of nine PVC/PVC-UV mixtures, respectively. It is clear that the spectra before and after UV irradiation were very similar without the significant change of peak of carbonyl during the photoaging process, which is completely different from PVC. The carbonyl index of all nine samples also ascended very slowly (Figs 13 and S11 (see supporting information), and Fig. S12 (see supporting information)), which was similar with pure PVC-UV without mixing. The carbonyl index of all nine PVC/PVC-UV mixtures after 200 h of UV irradiation are summarized in Table 1 and all values (up to 0.0991) are much lower than that of PVC (0.3331). It was found from Table 1 that the index decreased with the rising of the content of benzophenone moiety for the samples mixed with same PVC-UV. For the samples mixed with PVC-UV-7.5% or PVC-UV-12.9%, the carbonyl indexes were all below 0.05 (Table 1) and the lowest values were 0.0267 (PVC-UV-7.5%-Mix3) and 0.0231 (PVC-UV-12.9%-Mix3), respectively. These two similar values meant that the sample mixed with PVC-UV-7.5% could match that mixed with PVC-UV-12.9%. Thus, we can infer that PVC-UV-7.5% with moderate content of benzophenone moiety is the suitable polymeric UV-absorber for PVC matrix with great resistance to UV irradiation, low cost, and high processibilty.

**Figure 14:**
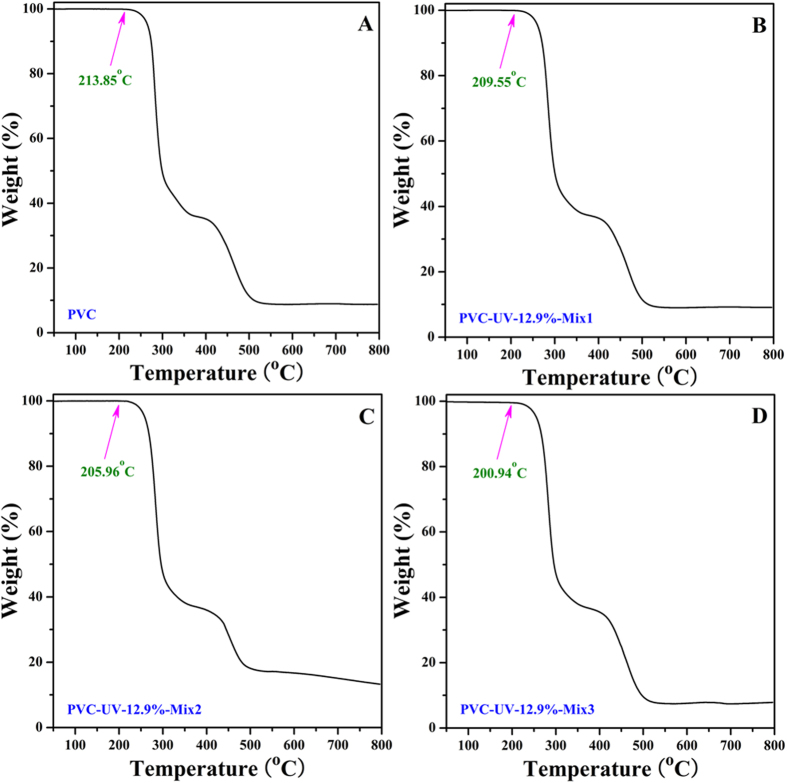
TGA (in N_2_) curves of pristine PVC (**A**), PVC-UV-12.9%-Mix1 (**B**), PVC-UV-12.9%-Mix2 (**C**), and PVC-UV-12.9%-Mix3 (**D**) with a heating rate of 10 ^o^C/min.

Furthermore, we examined the thermal stability of PVC/PVC-UV mixtures with different formulations by TGA (Fig. 14). PVC is generally used below 100 ^o^C and it began to degrade at 213.85 ^o^C in N_2_ as shown in Fig. 14A. The onset temperatures for degradation of three PVC/PVC-UV-12.9% mixtures with different compositions were in the range from 200 °C to 210 °C (Fig. 14B–D), just a very minor decreasing compared to that of pristine PVC. This result clearly indicated that PVC/PVC-UV mixture kept the thermal stability in comparison with pristine PVC.

## Conclusions

**Figure 15:**
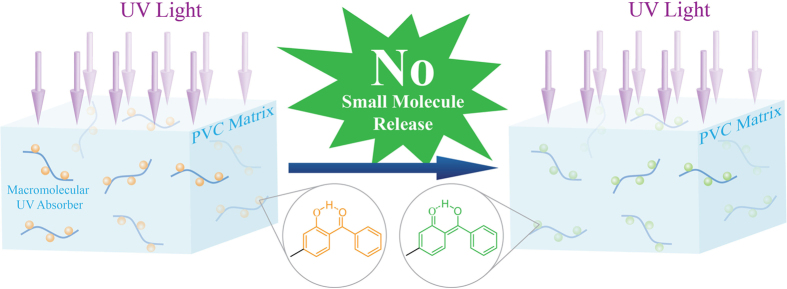
Schematic illustration of polymeric nontoxic additive strategy for stabilizing PVC against UV-photoaging without environment pollution.

In summary, we developed a polymeric nontoxic additive strategy to solve the puzzle of separation of small molecule additive for PVC (Fig. 15). The PVC-based UV-absorber was synthesized via copper-catalyzed Husigen-Click cycloaddition reaction under mild conditions with quantitative functionality conversion while keeping polymeric skeleton. The polymeric UV-absorber is soluble in common organic solvents and nontoxic *vs*. HaCat cell. They showed great resistance to UV irradiation (200 h) without photodegradation while no small molecule was released. Most importantly, the polymeric UV-absorber is compatible with PVC and could be used as additive to stabilize PVC against UV-photoaging without degradation even after 200 h of UV irradiation. The polymeric UV-absorber not only can be used as additive for PVC to greatly enhance the resistance to photoaging while keeping thermal stability, but can avoid the environment pollution via the separation of small molecule UV absorber. This is a breakthrough of additive for PVC to save PVC from the puzzle of separation of small molecule additive, and the great resistance to UV irradiation, low cost, and high processibilty of the polymeric UV-absorber are in favor of its industrial application. This strategy can be certainly extended to other type of additive for attaching them onto the main chain of PVC, to afford a multifunctional polymeric additive.

## Additional Information

**How to cite this article**: Huang, Z. *et al.* Construction of Nontoxic Polymeric UV-Absorber with Great Resistance to UV-Photoaging. *Sci. Rep.* **6**, 25508; doi: 10.1038/srep25508 (2016).

## Supplementary Material

Supplementary Information

## Figures and Tables

**Figure 1:**
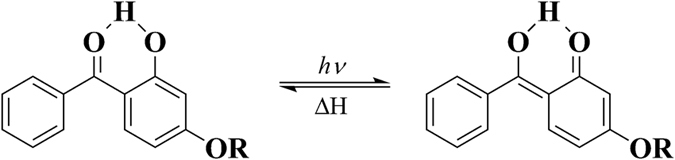
Transition of benzophenone UV-absorbers under UV light.

**Figure 2:**
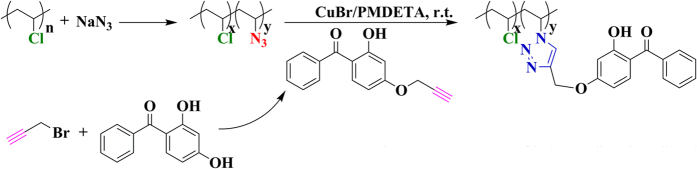
Synthesis of PVC-based UV-absorber.

**Table 1:** 

Sample	*M*_n_^a^ (g/mol)		*M*_w_/*M*_n_^a^		Carbonyl index^b^
	0 h	200 h	0 h	200 h	
PVC-UV-3.5%-Mix1	46,400	45,700	1.62	1.65	0.0991
PVC-UV-3.5%-Mix2	46,800	45,900	1.62	1.64	0.0778
PVC-UV-3.5%-Mix3	47,300	46,700	1.60	1.61	0.0624
PVC-UV-7.5%-Mix1	48,100	47,700	1.60	1.61	0.0476
PVC-UV-7.5%-Mix2	48,700	48,100	1.58	1.60	0.0339
PVC-UV-7.5%-Mix3	49,500	49,100	1.62	1.62	0.0267
PVC-UV-12.9%-Mix1	48,800	48,300	1.58	1.59	0.0314
PVC-UV-12.9%-Mix2	49,300	48,800	1.57	1.56	0.0305
PVC-UV-12.9%-Mix3	50,400	50,000	1.58	1.58	0.0231

Molecular Weight and Carbonyl Index of PVC/PVC-UV Mixtures.

^a^Measured by GPC in THF at 35 ^o^C.

^b^Determined by FT-IR.
