# Supplementary material for: Construction of Nontoxic Polymeric UV-Absorber with Great Resistance to UV-Photoaging
Source: Sci Rep. 2016 May 3;6:25508. doi: 10.1038/srep25508 (PMC4853786; doi:10.1038/srep25508)
Supplement: Supplementary Information [file srep25508-s1.doc]

**Supporting information for**

**Construction of Nontoxic Polymeric UV-Absorber with Great Resistance to UV-Photoaging**

*Zhong Huang*,*^1,#^ Aishun Ding*,*^1,2,#^ Hao Guo*,*^2^,** *Guolin Lu*,*^1^ Xiaoyu Huang^1^,**

^1^ Key Laboratory of Synthetic and Self-Assembly Chemistry for Organic Functional Molecules, Shanghai Institute of Organic Chemistry, Chinese Academy of Sciences, 345 Lingling Road, Shanghai 200032, People’s Republic of China

^2^ Department of Chemistry, Fudan University, 220 Handan Road, Shanghai 200433, People’s Republic of China

**Experimental Section**

**Materials**

Poly(vinyl chloride) (PVC, *M*_n_ = 46,000 g/mol, *M*_w_/*M*_n_ = 1.62, *T*_g_: 78.22^o^C) was donated by China Petrochemical Corporation. Copper(I) bromide (CuBr, Aldrich, 98%) was purified by stirring overnight over CH_3_COOH at room temperature, followed by washing with ethanol, diethyl ether, and acetone prior to drying at 40^o^C *in vacuo* for one day. Tetrahydrofuran (THF, Aldrich, 99%) was dried over CaH_2_ and distilled from Na and benzophenone under N_2_ prior to use. Resorcinol (Aldrich, 99%), benzoic acid (Aldrich, 99.5%), 3-bromo-1-propyne (Aldrich, 99%), sodium azide (NaN_3_, Aldrich, 99%), tetra-*n*-octylammonium bromide (TOAB, Aldrich, 98%), *N,N,N*’*,N*’*,N’*’-pentamethyldiethylenetriamine (PMDETA, Aldrich, 99%), and potassium carbonate (K_2_CO_3_, Aldrich, 99%) were used as received. 2,4-Dihydroxybenzophenone was prepared according to previous literature^1^ using resorcinol and benzoic acid as starting materials.

**Measurements**

FT-IR spectra were recorded on a Nicolet AVATAR-360 FT-IR spectrophotometer with a resolution of 4 cm^-1^. ^1^H NMR analyses were performed on a Bruker Avance 500 spectrometer (500 MHz) in CDCl_3_, tetramethylsilicone was used as internal standard. Relative molecular weights and molecular weight distributions were measured by conventional gel permeation chromatography (GPC) system equipped with a Waters 1515 Isocratic HPLC pump, a Waters 2414 refractive index detector, a Waters 2487 dual λ absorbance detector, and a set of Waters Styragel columns (HR3 (500-30,000), HR4 (5,000-600,000) and HR5 (50,000-4,000,000), 7.8×300 mm, particle size: 5 μm). GPC measurements were carried out at 35^o^C using THF as eluent with a flow rate of 1.0 mL/min. The system was calibrated with linear polystyrene standards. UV/vis spectra were measured by a Hitachi U-2910 [spectrophotometer](javascript:popupOBO('CMO:0002232','c2py20933k')) with a rate of 200 nm/min. Differential scanning calorimetry (DSC) measurements were run on a TA Q200 system under N_2_ purge with a heating rate of 10^o^C/min. The glass transition temperature (*T*_g_) was recorded from the second heating process after a quick cooling from 200^o^Cand the value was determined from the midpoint of *C*_p_ curve. Thermogravimetry analysis (TGA) measurements were run on a TA Q500 system under N_2_ purge with a heating rate of 10^o^C/min. Elemental analysis was carried out on a Carlo-Erba1106 system.

**Propargylation of 2,4-dihydroxybenzophenone**

2,4-Dihydroxybenzophenone (25.2 g, 0.118 mol) and K_2_CO_3_ (32.5 g, 0.236 mol) were first dissolved in acetone (300 mL) followed by adding 3-bromo-1-propyne (9.2 mL, 0.118 mol). The mixture was refluxed for 16 h and a white powder, (2-hydroxy-4-(prop-2-ynyloxy)phenyl)(phenyl)methanone (22.3 g, 75% yield), was obtained from column chromatography. ^1^H NMR: *δ* (ppm): 2.59 (1H, OCH_2_C≡C*H*), 4.76 (2H, OC*H*_2_C≡CH), 6.50, 6.63, 7.54, 7.64 (8H, phenyl), 12.67 (1H, O*H*). UV-vis: λ (nm): 210, 241, 284, 326.

**Substitution of PVC with NaN_3_**

PVC (*M*_n_ = 46,000 g/mol, *M*_w_/*M*_n_ = 1.62, 3.00 g, 0.048 mol Cl), NaN_3_ (3.80 g, 0.058 mol), and the phase transfer catalyst, TOAB (0.26 g, 0.0048 mol), were added 30 mL of freshly-distilled THF. The mixture was stirred at 50^o^C for a period of time followed precipitating the mixture into CH_3_OH/H_2_O (v:v = 2:1). The crude product was purified by repeated dissolution and precipitation followed by drying *in vacuo* overnight to give the product of PVC-N_3_. FT-IR (KBr): *ν* (cm^-1^): 2973, 2910, 2863, 2115 (-N_3_), 1429, 1356, 1336, 1254, 1066, 965, 909, 693, 615. ^1^H NMR: *δ* (ppm): 1.58, 1.69, 1.87, 2.08 (2H, C*H*_2_CHCl and C*H*_2_CHN_3_), 3.78, 3.96, 4.19 (1H, CH_2_C*H*N_3_), 4.34, 4.48, 4.60 (1H, CH_2_C*H*Cl).


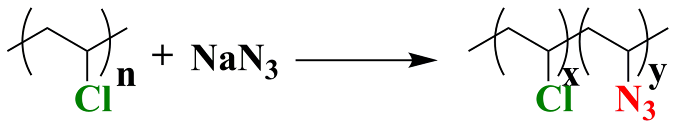


**x + y = 1 (1)**

**42y/(62.5x + 69y) = N% (2)**

The substitution ratio of -N_3_ was tuned by varying reaction time and was determined by elemental analysis according to eq. 1 and 2 as summarized in Table S1.

**Table S1.** Characterization of PVC-N_3_

| Entry | Reaction time (h) | N%^a^ | N_3_%^b^ | *M*_n_^c^ (g/mol) | *M*_w_/*M*_n_^c^ |
| --- | --- | --- | --- | --- | --- |
| 1 | 4.0 | 2.35 | 3.5 | 47,200 | 1.56 |
| 2 | 12.0 | 5.00 | 7.5 | 47,800 | 1.55 |
| 3 | 14.0 | 8.64 | 12.9 | 48,600 | 1.58 |

^a^ Measured by elemental analysis. ^b^ Determined by elemental analysis according to eq. 1 and 2. ^c^ Measured by GPC at 35^o^C in THF.

**Click reaction between (2-hydroxy-4-(prop-2-ynyloxy)phenyl)(phenyl)methanone and PVC-N_3_**

In a typical procedure, PVC-N_3_-7.5% (*M*_n_ = 47,800 g/mol, *M*_w_/*M*_n_ = 1.55, 2.60 g, 4.64 mmol N_3_), (2-hydroxy-4-(prop-2-ynyloxy)phenyl)(phenyl)methanone (1.23 g, 4.88 mmol), and CuBr (134 mg, 0.93 mmol) were first added to a 100 mL Schlenk flask (flame-dried under vacuum prior to use) sealed with a rubber septum for degassing and kept under N_2_. Next, PMDETA (0.194 mL, 0.93 mmol) and freshly- distilled THF (40 mL) were added via a gastight syringe. The flask was degassed by three cycles of freezing-pumping-thawing. The reaction lasted 10 h at room temperature. The reaction mixture was diluted with THF and filtered through the neutral aluminum oxide column. The solution was precipitated into methanol. The crude product was purified by repeated dissolution and precipitation followed by drying *in vacuo* overnight to give 2.85 g of white powder, PVC-UV-7.5%. GPC: *M*_n_ = 55,400 g/mol, *M*_w_/*M*_n_ = 1.57. FT-IR (KBr): *ν* (cm^-1^): 3147, 3061, 2969, 2910, 1623, 1576, 1502, 1429, 1341, 1255, 1190, 1166, 1111, 1044, 1010, 965, 837, 804, 703, 615. ^1^H NMR: *δ* (ppm): 1.65, 2.06, 2.29 (2H, C*H*_2_CH), 3.46, 3.60 (1H, CH_2_C*H*N), 4.29, 4.44, 4.57 (1H, CH_2_C*H*Cl), 5.20, 5.29 (2H, OC*H*_2_), 6.49, 6.63, 7.51, 7.62 (8H, phenyl), 7.79 (1H, =C*H* of triazole ring), 12.65 (1H, O*H*).

**Table S2.** Characterization of PVC-UV

| Sample | *M*_n_^a^ (g/mol) | *M*_w_/*M*_n_^a^ | *T*_g_^b^ (^o^C) |
| --- | --- | --- | --- |
| PVC-UV-3.5% | 51,800 | 1.56 | 81.35 |
| PVC-UV-7.5% | 55,400 | 1.57 | 82.02 |
| PVC-UV-12.9% | 57,700 | 1.59 | 82.93 |

^a^ Measured by GPC at 35^o^C in THF. ^b^ Measured by DSC from the second heating process under N_2_ purge with a heating rate of 10^o^C/min.

**Mixing PVC-UV with PVC**

In a typical procedure, PVC-UV-7.5% (*M*_n_ = 55,400 g/mol, *M*_w_/*M*_n_ = 1.57, 0.10 g, 0.0916 mol benzophenone moiety) and PVC (*M*_n_ = 46,000 g/mol, *M*_w_/*M*_n_ = 1.62, 1.90 g) were first dissolved in 20 mL of freshly-distilled THF. The solution was stirred at room temperature for 1 h and precipitated into methanol. The crude product was purified by repeated dissolution and precipitation followed by drying *in vacuo* overnight to give 1.87 g of white powder, PVC-UV-7.5%-Mix1. GPC: *M*_n_ = 48,100 g/mol, *M*_w_/*M*_n_ = 1.60. The formulations of all nine mixtures are listed in Table S3.

**Table S3.** Formulation of PVC/PVC-UV Mixtures

| sample | PVC/PVC-UV | *W*_benzophe_ (g) | *n*_benzophe_ (mol) |
| --- | --- | --- | --- |
| PVC-UV-3.5%-Mix1 | 1.8125 g PVC +  0.1875 g PVC-UV-3.5% | 0.0231 | 9.16×10^-5^ |
| PVC-UV-3.5%-Mix2 | 1.6250 g PVC +  0.3750 g PVC-UV-3.5% | 0.0462 | 1.83×10^-4^ |
| PVC-UV-3.5%-Mix3 | 1.4375 g PVC +  0.5625 g PVC-UV-3.5% | 0.0693 | 2.75×10^-4^ |
| PVC-UV-7.5%-Mix1 | 1.9000 g PVC +  0.1000 g PVC-UV-7.5% | 0.0231 | 9.16×10^-5^ |
| PVC-UV-7.5%-Mix2 | 1.8000 g PVC +  0.2000 g PVC-UV-7.5% | 0.0462 | 1.83×10^-4^ |
| PVC-UV-7.5%-Mix3 | 1.7000 g PVC +  0.3000 g PVC-UV-7.5% | 0.0693 | 2.75×10^-4^ |
| PVC-UV-12.9%-Mix1 | 1.9320 g PVC +  0.0680 g PVC-UV-12.9% | 0.0231 | 9.16×10^-5^ |
| PVC-UV-12.9%-Mix2 | 1.8640 g PVC +  0.1360 g PVC-UV-12.9% | 0.0462 | 1.83×10^-4^ |
| PVC-UV-12.9%-Mix3 | 1.7980 g PVC +  0.2040 g PVC-UV-12.9% | 0.0693 | 2.75×10^-4^ |

**Ultraviolet aging test**

The photostability of PVC, PVC-UV with different amount of benzophenone moiety, and PVC/PVC-UV mixture with different formulation was compared by exposing them in a UV-aging box for 200 h in powder state 20^o^C. PVC is very sensitive to 310 nm UV light so that a UV-aging box (110 W) with a 313 nm UV lamp was selected as the light source.

During the light aging process, a sample was taken every 10 h, and the photodegradation was monitored by testing the color change^2^ and the carbonyl index change.^3,4^ Carbonyl index was defined as the integration area ratio of carbonyl peak (around 1700 cm^-1^) to methylene peak (around 1430 cm^-1^). Carbonyls were formed during the photodegradation and usually used as the main index of photodegradation.

***in vitro* Cytotoxicity**

The cytotoxicity of PVC, PVC-UV, and PVC/PVC-UV mixture was tested by following methods.

The human immortalized non-tumorigenic keratinocyte cell line HaCaT was supplied by American Type Culture Collection (ATCC, USA). They were cultured at 37^o^C under a humidified 5% CO_2_ atmosphere in DMEM medium (GIBCO/Invitrogen, USA) supplemented with 10% fetal bovine serum (FBS, BI Biological Industries Ltd., Israel) and 1% penicillin-streptomycin (10,000 U/mL penicillin and 10 mg/mL streptomycin, Solarbio life science, China).

Cell viability was evaluated by Cell Counting Kit-8 (CCK-8, Dojindo, Japan) according to the factory’s instruction. HaCat Cells were plated in 96 well plates at a density of 1×10^5^ cells per well in 100 μL of DMEM medium and added with desired concentrations of impregnated solutions from different materials. The relative cell viability was measured by Cell Counting Kit-8 (Dojindo, Japan). After continuous incubation for 72 h, absorbance was measured at 450 nm with a Tecan Genios multifunction-reader.

**References and Notes**

1. Sharghi, H.; Hosseini-Sarvari, M.; Eskandari, R. *Synthesis* **2006**, 2047.
2. Bock, V. D.; Heimsha, H.; van Morsteen, S. H. *Eur*. *J*. *Org*. *Chem*. **2006**, *51*, 2606.
3. Sombatsompop, N. *J*. *Appl*. *Polym*. *Sci*. **2004**, *92*, 84.
4. Jerzy, W. In *Polyvinyl Chloride Degradation*; Elsevier Science Publishing Company: New York, 1985; p 243.


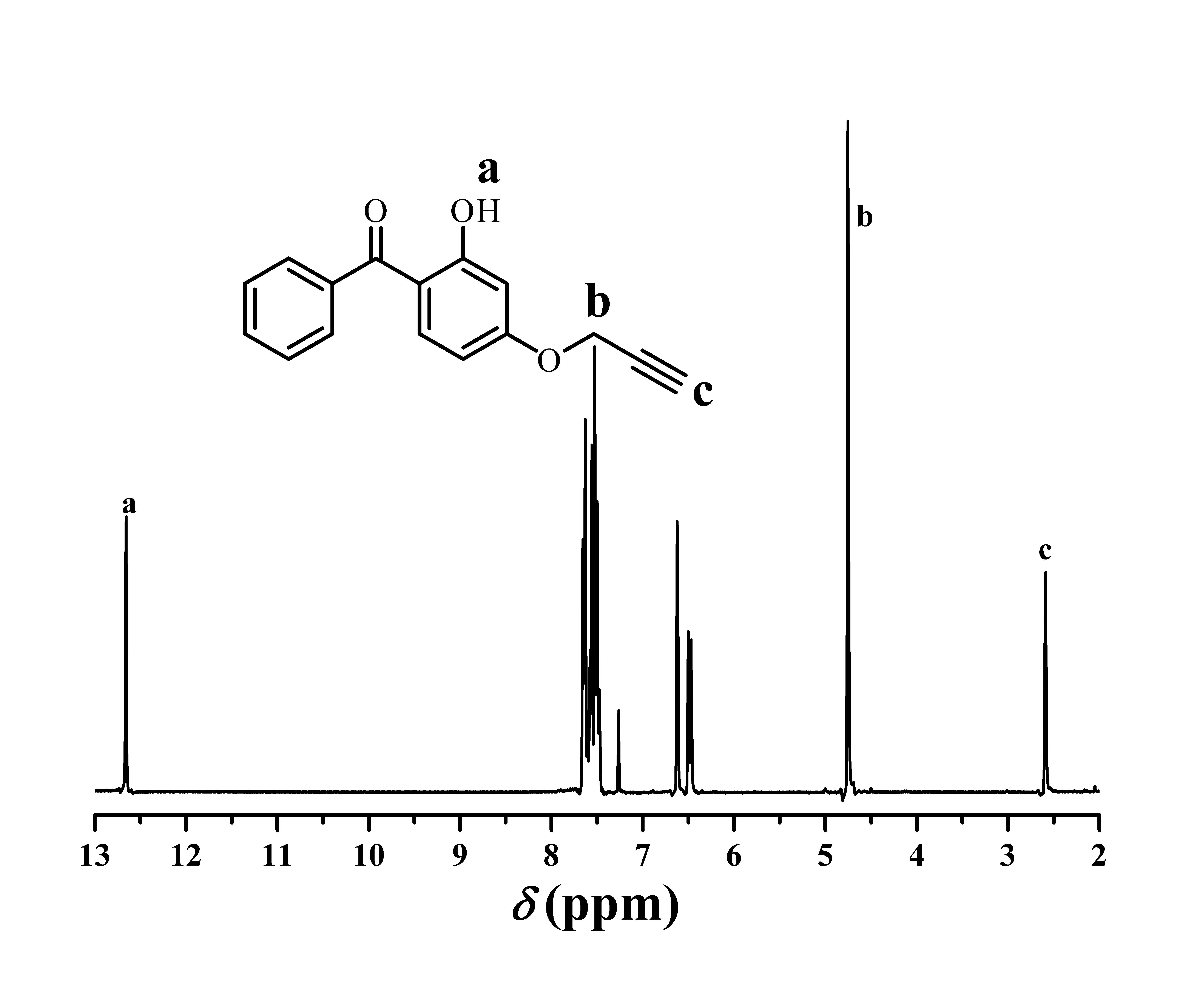


**Figure S1.** ^1^H NMR spectrum of (2-hydroxy-4-(prop-2-ynyloxy)phenyl)(phenyl)- methanone in CDCl_3_.


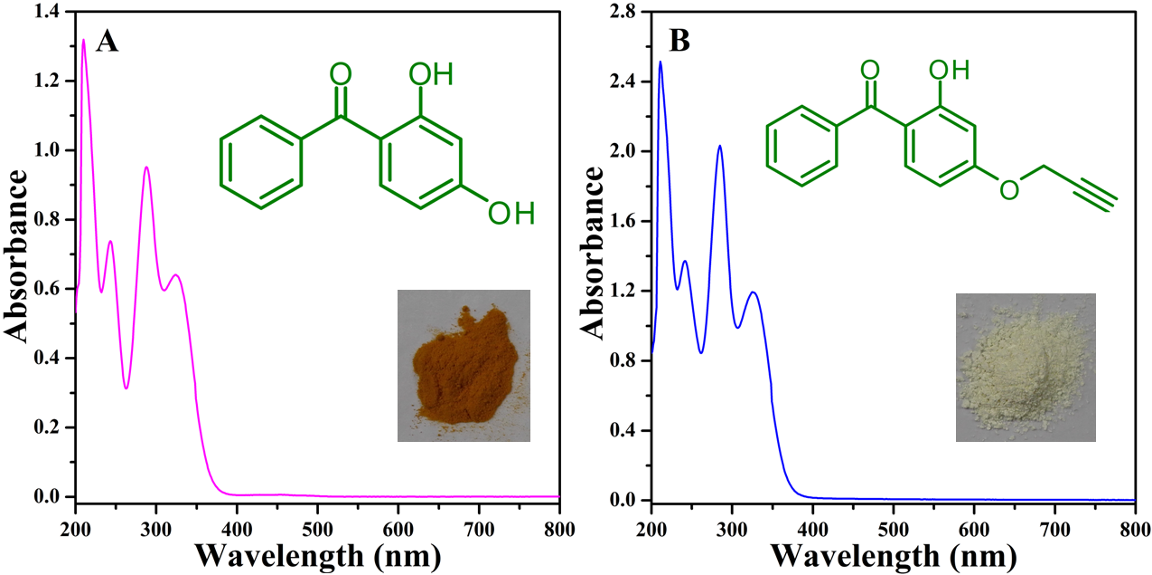


**Figure S2.** UV-vis spectra of 2,4-dihydroxybenzophenone (A) and (2-hydroxy-4- (prop-2-ynyloxy)phenyl)(phenyl)methanone (B) in THF.


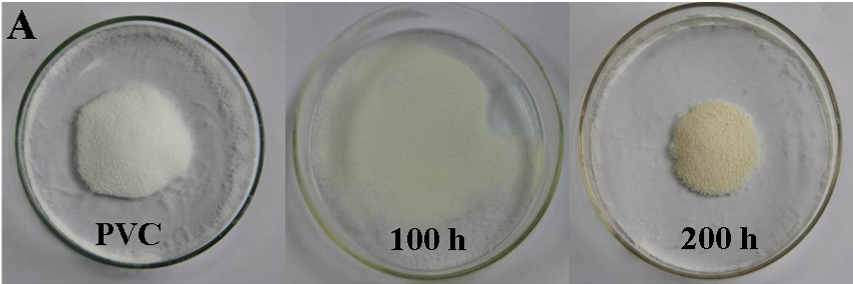


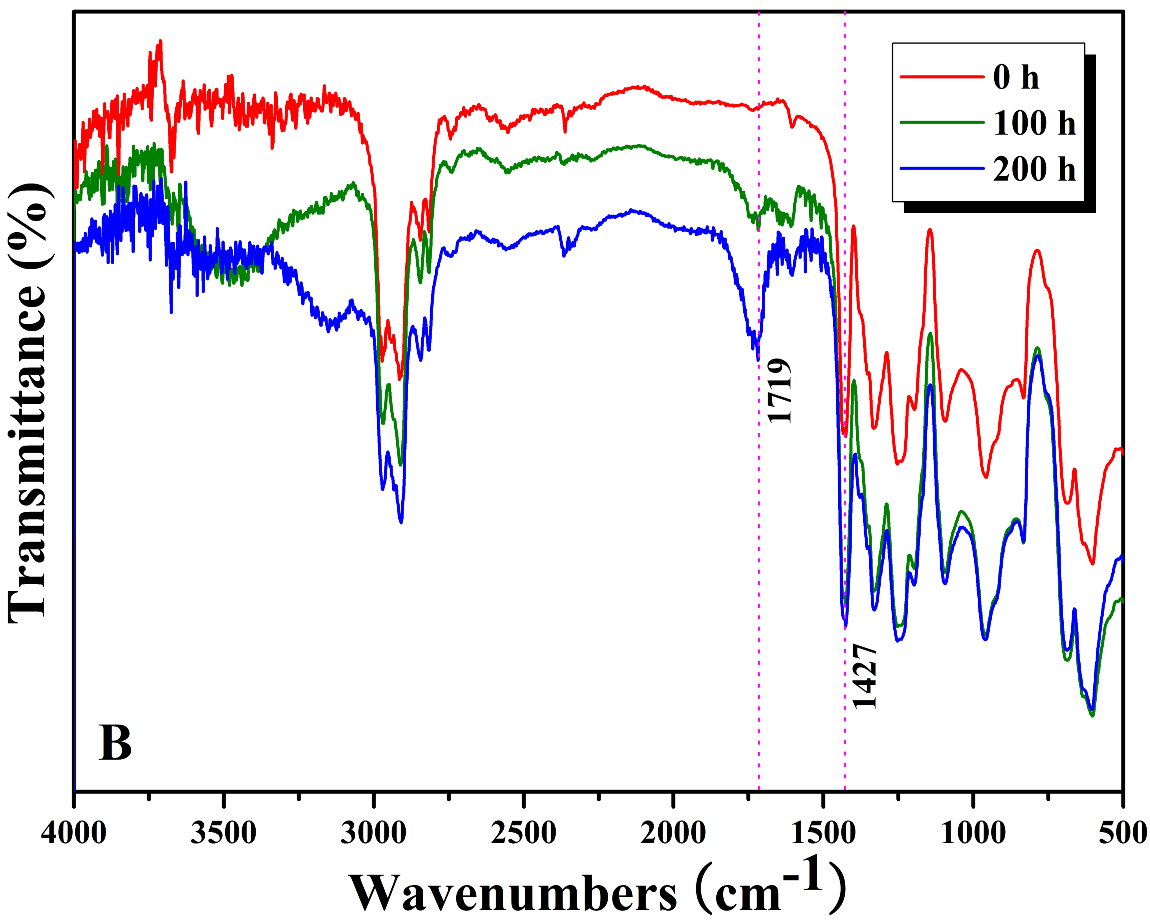


**Figure S3.** (A) Color changes of PVC under UV irradiation. (B) FT-IR spectra of PVC under UV irradiation.


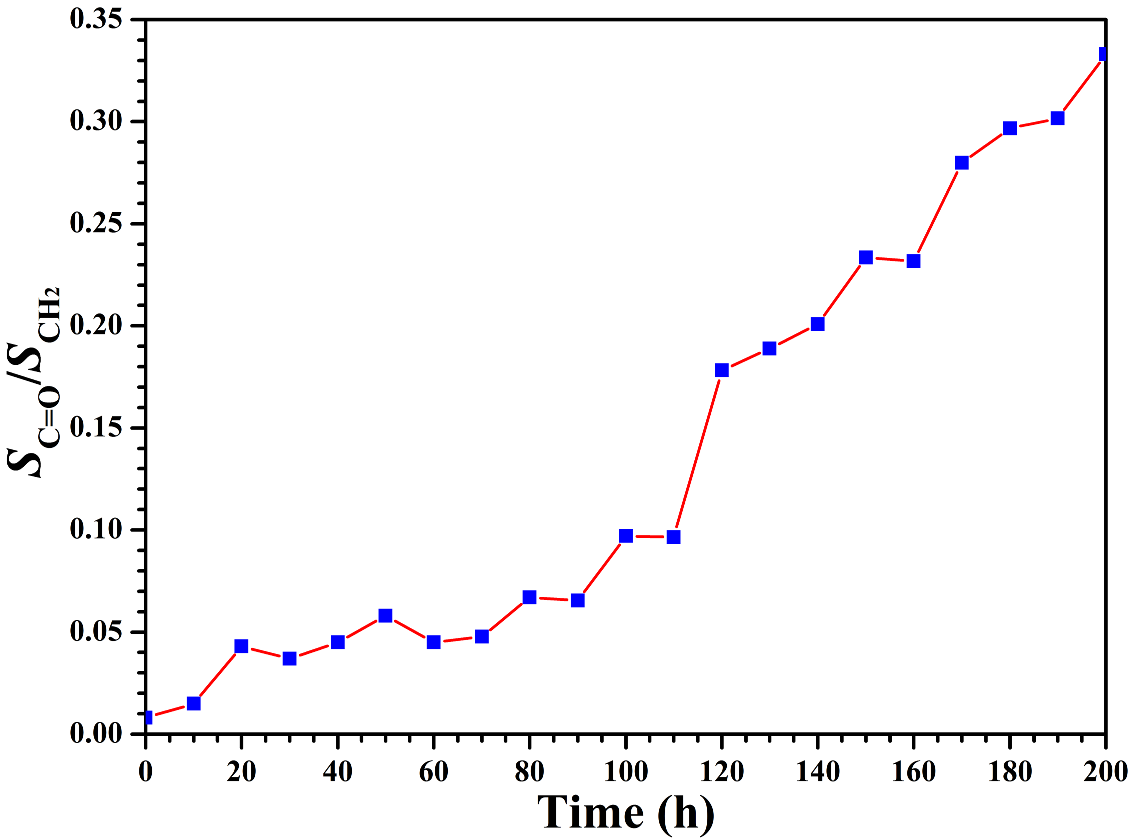


**Figure S4.** Dependence of the integration area ratio of carbonyl peak to methylene peak (*S*_C=O_/*S*_CH2_) of PVC on UV irradiation time at 20^o^C.


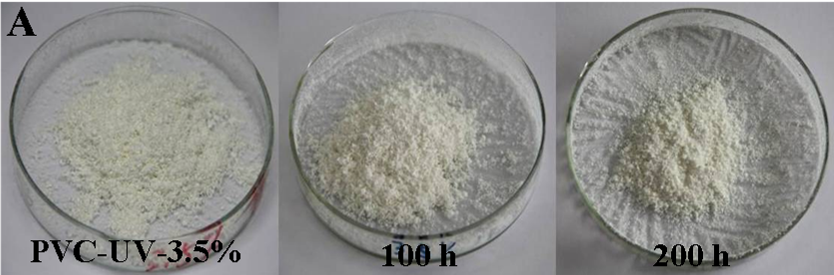


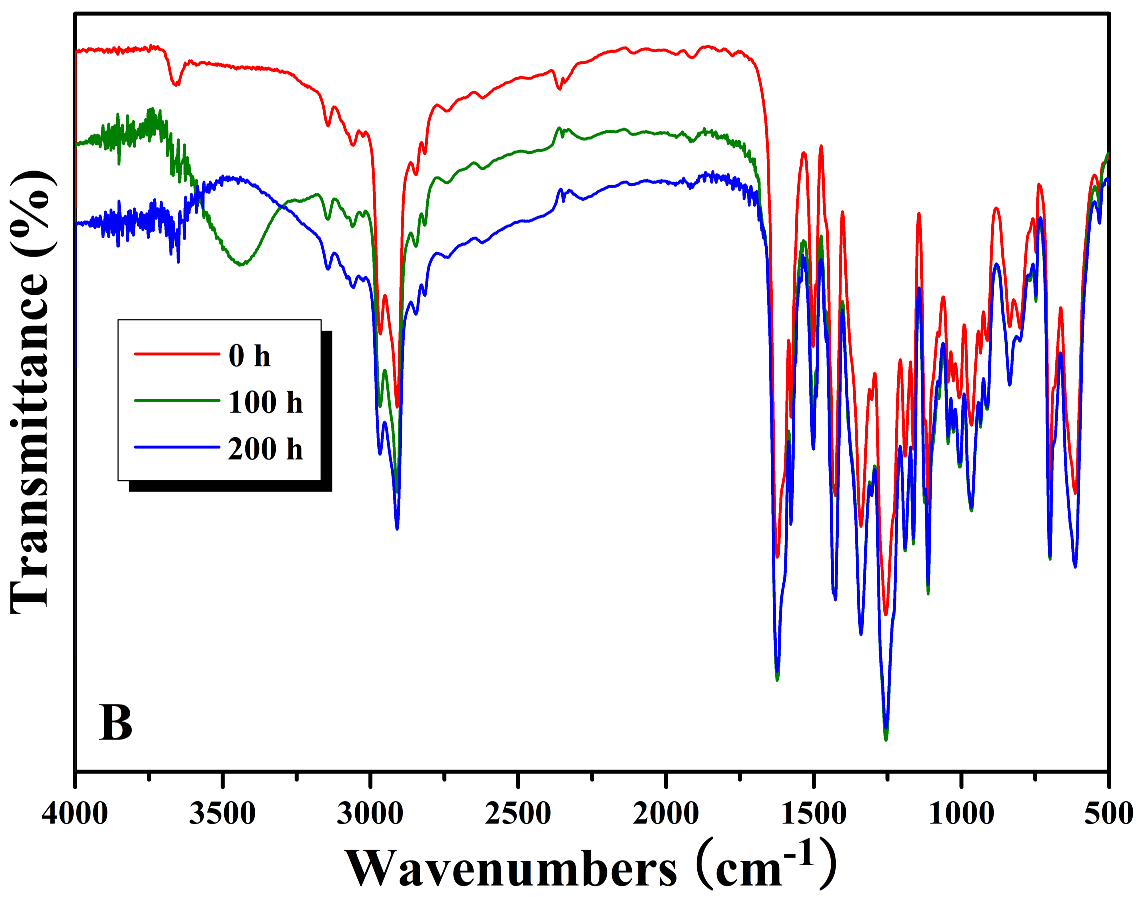


**Figure S5.** (A) Color changes of PVC-UV-3.5% under UV irradiation. (B) FT-IR spectra of PVC-UV-3.5% under UV irradiation.


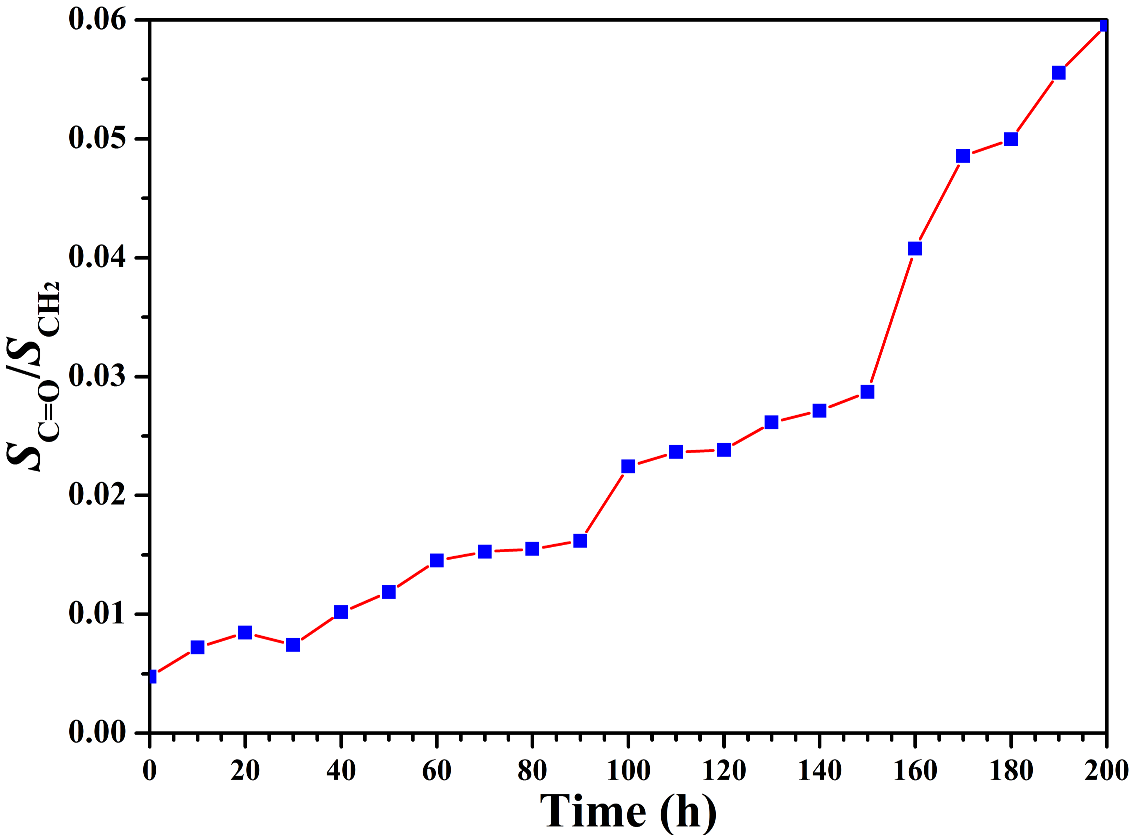


**Figure S6.** Dependence of the integration area ratio of carbonyl peak to methylene peak (*S*_C=O_/*S*_CH2_) of PVC-UV-3.5% on UV irradiation time at 20^o^C.


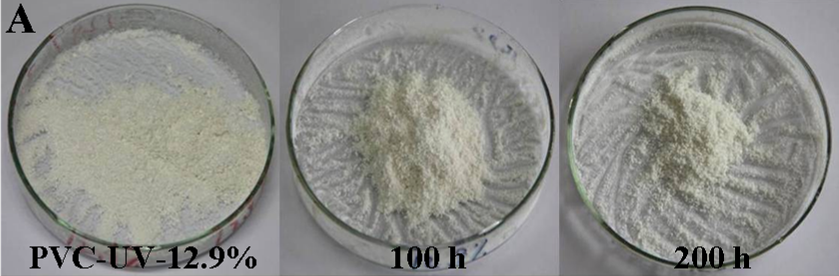


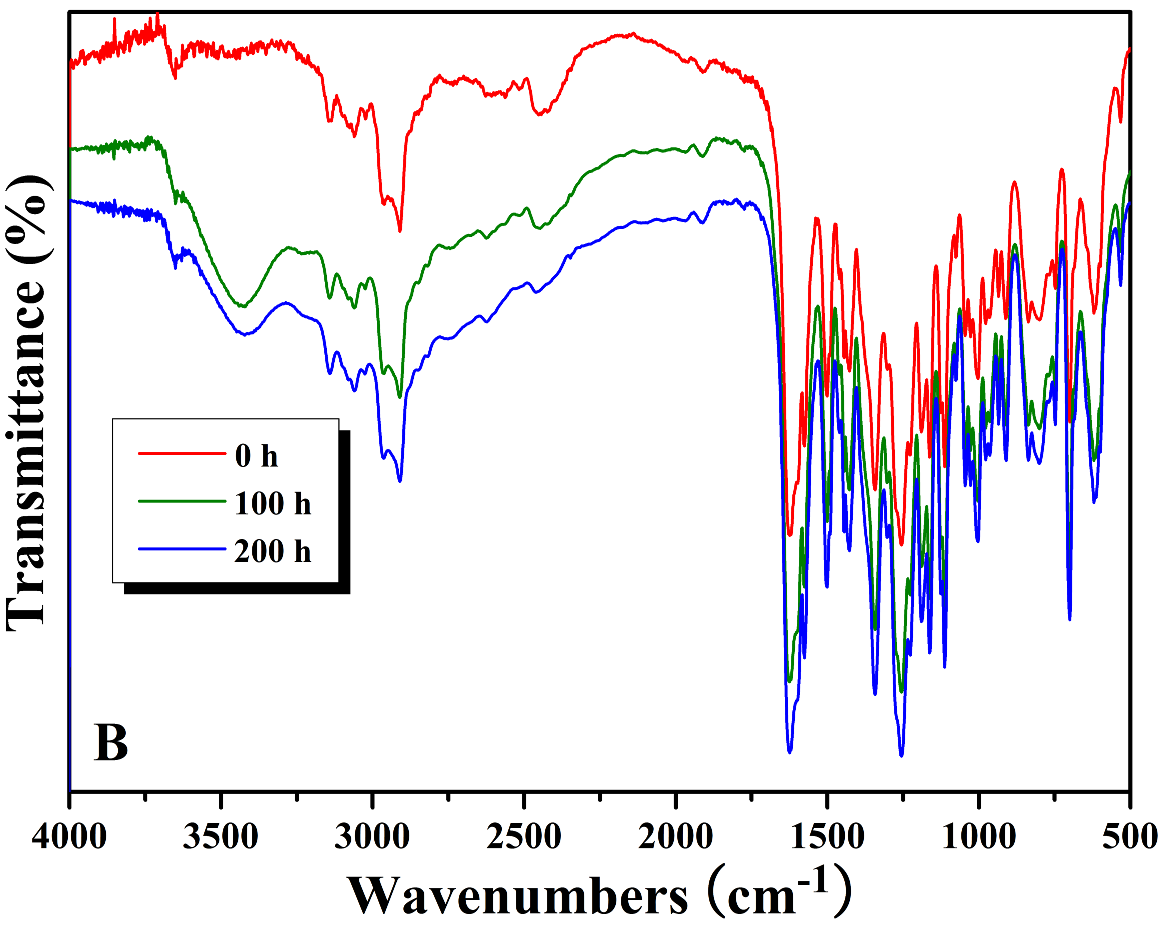


**Figure S7.** (A) Color changes of PVC-UV-12.9% under UV irradiation. (B) FT-IR spectra of PVC-UV-12.9% under UV irradiation.


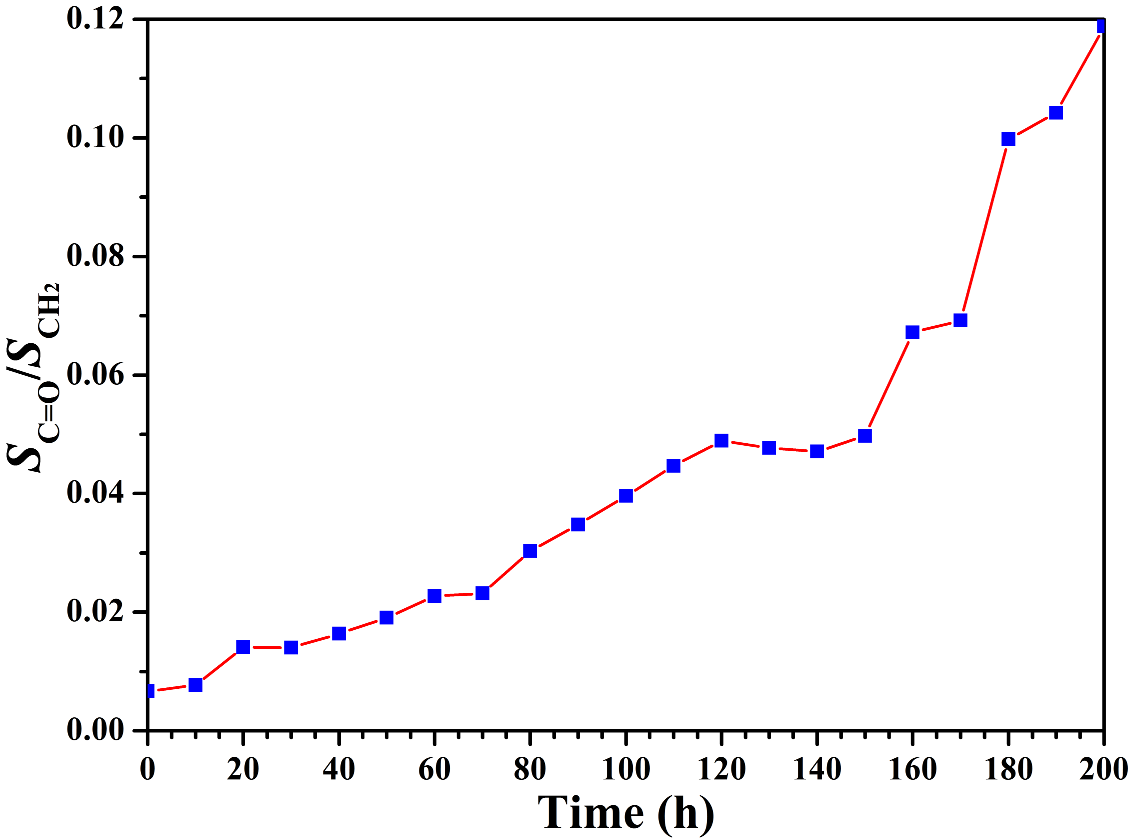


**Figure S8.** Dependence of the integration area ratio of carbonyl peak to methylene peak (*S*_C=O_/*S*_CH2_) of PVC-UV-12.9% on UV irradiation time at 20^o^C.


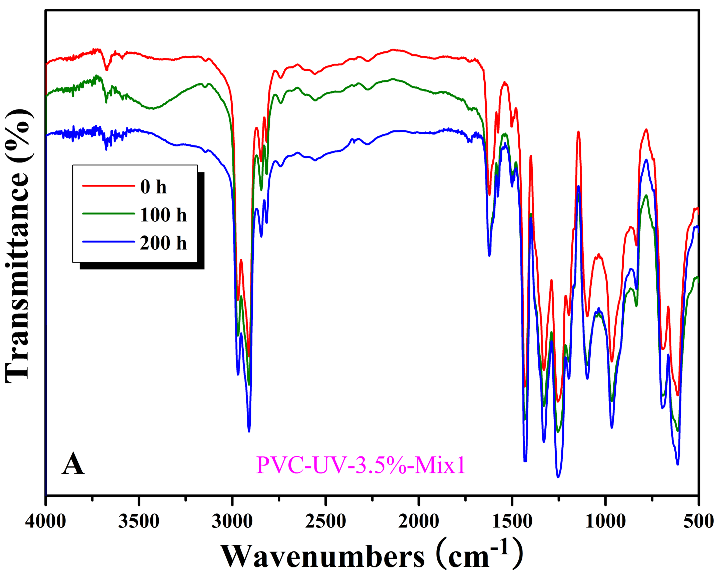


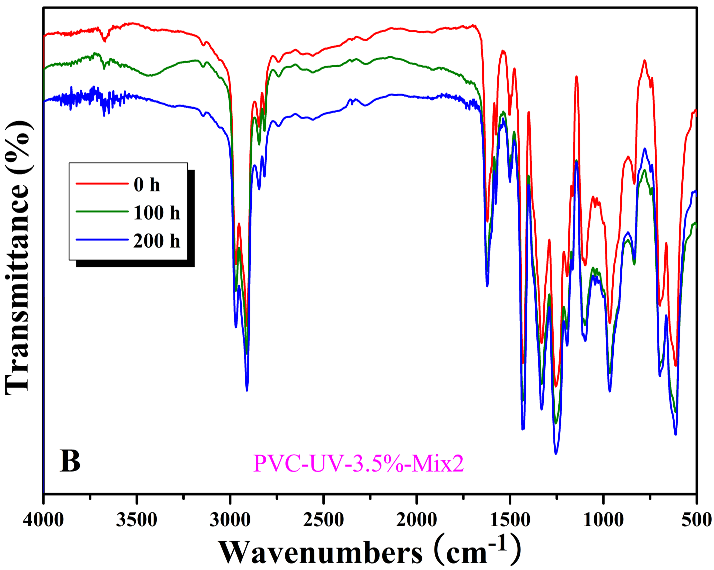


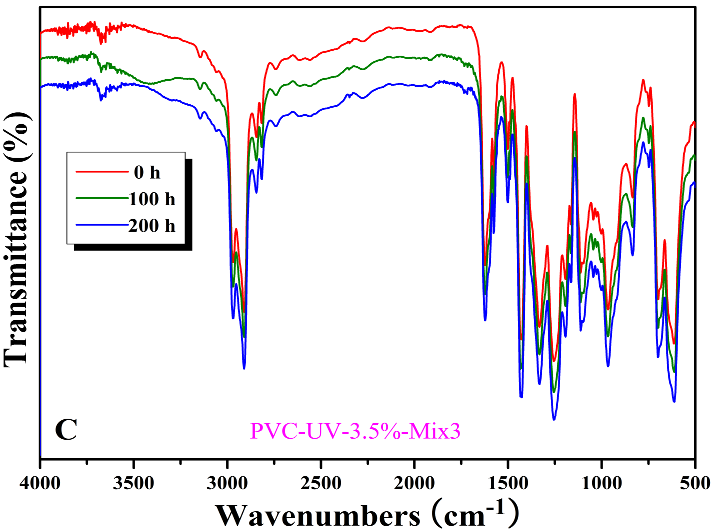


**Figure S9.** FT-IR spectra of PVC/PVC-UV-3.5% mixtures with different formulations under UV irradiation.


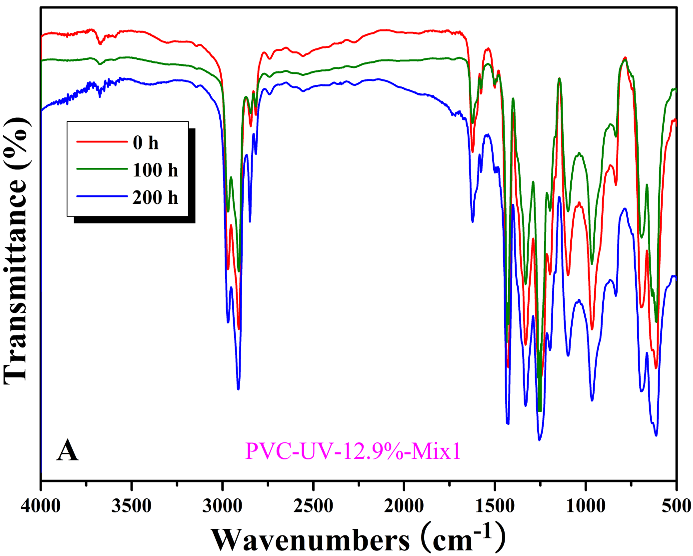


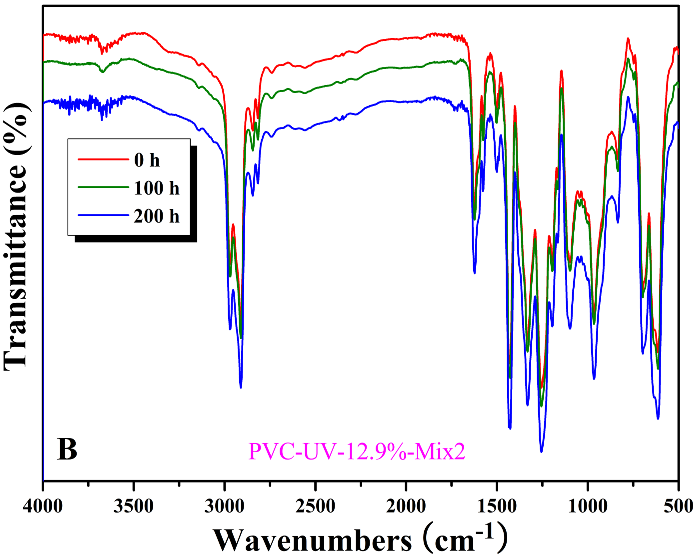


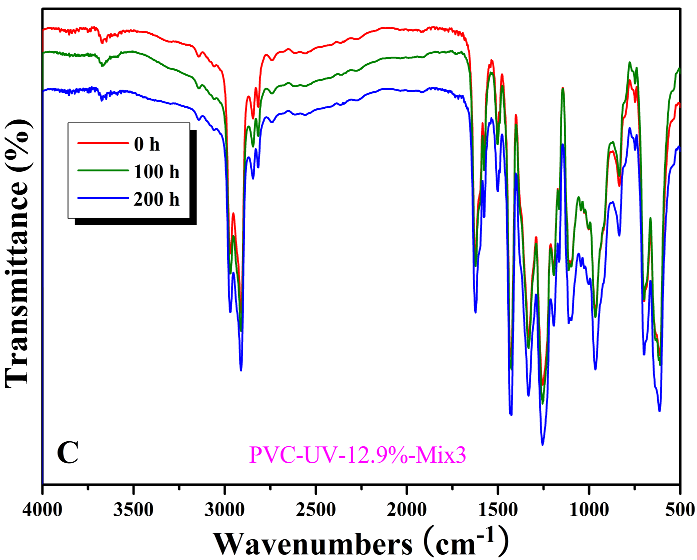


**Figure S10.** FT-IR spectra of PVC/PVC-UV-12.9% mixtures with different formulations under UV irradiation.


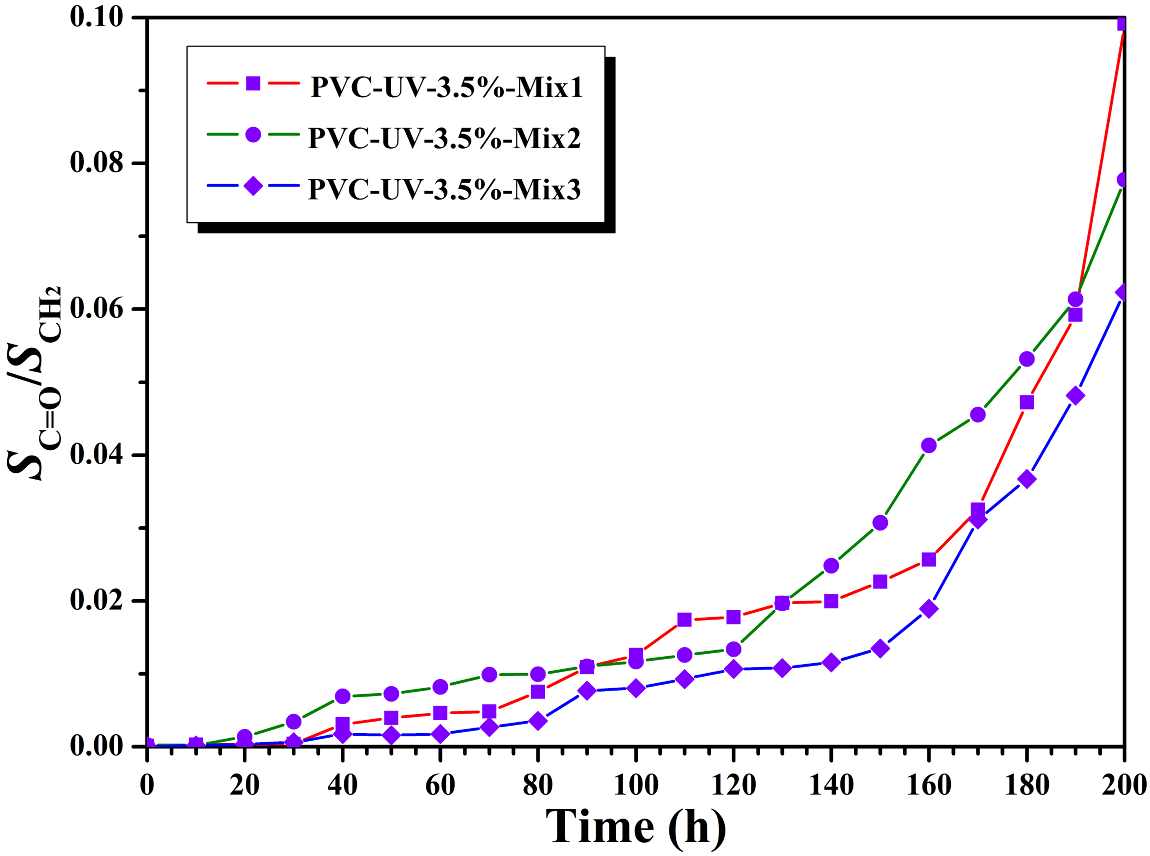


**Figure S11.** Dependence of the integration area ratio of carbonyl peak to methylene peak (*S*_C=O_/*S*_CH2_) of PVC/PVC-UV-3.5% mixtures with different formulations on UV irradiation time at 20^o^C.


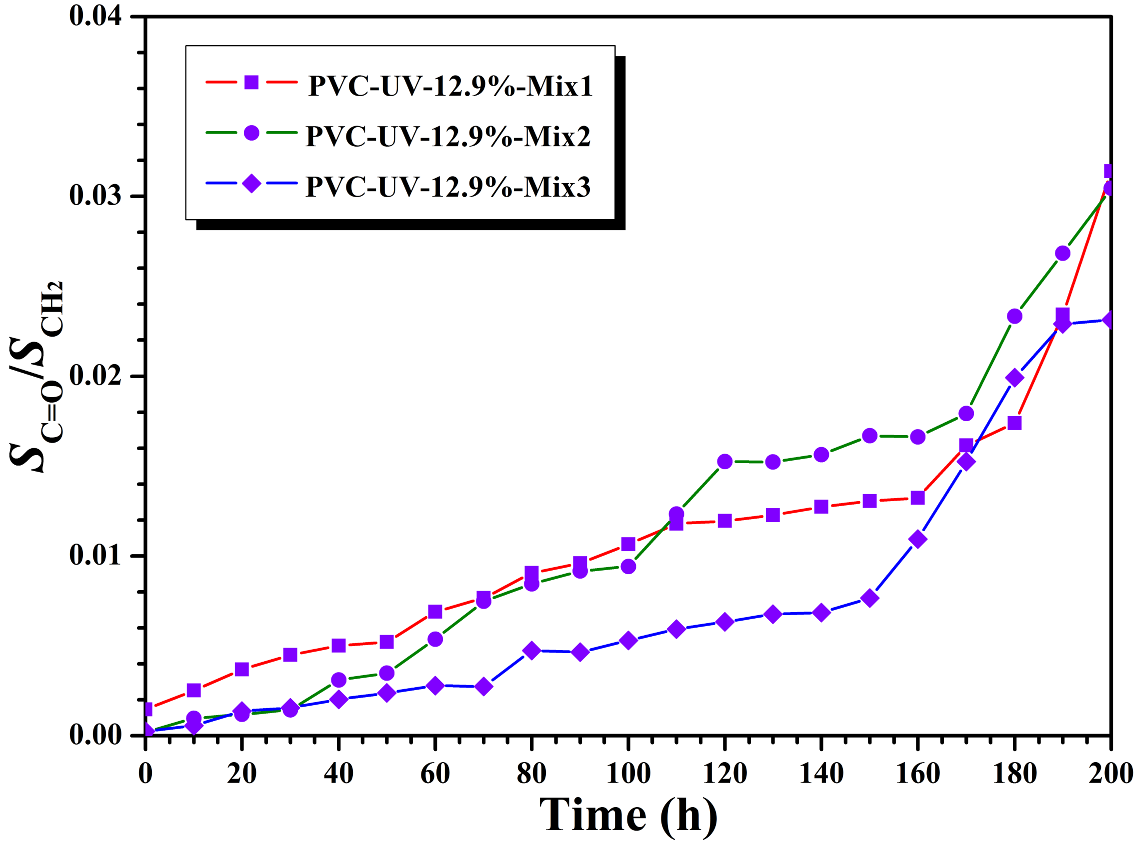


**Figure S12.** Dependence of the integration area ratio of carbonyl peak to methylene peak (*S*_C=O_/*S*_CH2_) of PVC/PVC-UV-12.9% mixtures with different formulations on UV irradiation time at 20^o^C.
